# Supplementary material for: Gold Nanocone Array with Extensive Electromagnetic Fields for Highly Reproducible Surface-Enhanced Raman Scattering Measurements
Source: Micromachines (Basel). 2022 Jul 27;13(8):1182. doi: 10.3390/mi13081182 (PMC9332797; doi:10.3390/mi13081182)
Supplement: Supplementary file 1 [file micromachines-13-01182-s001.zip › micromachines-1824510-supplementary.pdf]

Supplementary information

Gold Nanocone Array with Extensive Electromagnetic Fields for Highly-Reproducible Surface-Enhanced Raman Scattering Measurements

Satoko Fujiwara [1], Daiki Kawasaki [1], Kenji Sueyoshi [1,2], Hideaki Hisamoto [1], and Tatsuro Endo [1*]

1. Department of Applied Chemistry, Graduate School of Engineering, Osaka Metropolitan University, 1-1 Gakuencho, Nakaku, Sakai, Osaka 599-8531, Japan.
2. Japan Science and Technology Agency (JST), Precursory Research for Embryonic Science and Technology (PRESTO), 4-1-8 Honcho, Kawaguchi, Saitama 332-0012, Japan.

Explanation about FDTD simulation in this study
Figure S1: EM simulation using the FDTD method
Figure S2: Surface structure of the COP film as observed by FE-SEM and AFM
Figure S3: AFM images and cross-section of the surface structure of the COP film and Au NCA
Evaluation of the individual cone apex radii of the fabricated Au NCA and the COP film
Figure S4: EM field intensity distribution, absorption spectra, and maximum value of EM field intensities ($|E_{max}/E_0|$) of Au NCA with various thicknesses of the Au layer
Figure S5: SERS spectra of 10^{-6} M 4-MBA on Au NCA and raman spectra 10^{-2} M 4-MBA on Au glass.
Table S1: Band attribution of detected 4-MBA Raman spectra
Calculation of enhancement factor (EF)

Explanation about FDTD simulation in this study

The FDTD structure model (Figure S1) was mostly constructed to the results observed by SEM and AFM images of the fabricated Au NCA (Figure 2b, 2c and Figure S3). In this simulation, the polarized incident light was irradiated, which was different from the experiment. However, the optical properties were expected to be characterized because Au NCA was isotropic and LSPR mode did not depend on the lattice structure[42, 43]. We used a parabola cone (diameter: 300 nm, height: 200 nm) as the nanocone structures, which is matched with the images observed by SEM and AFM. But, in this study, the bottom of the nanocone structure was 100 nm below the surface of the Au plate because the size of NCA structure was different from the fabricated one than expected and the simulation results was actually not matched to the experimental one.

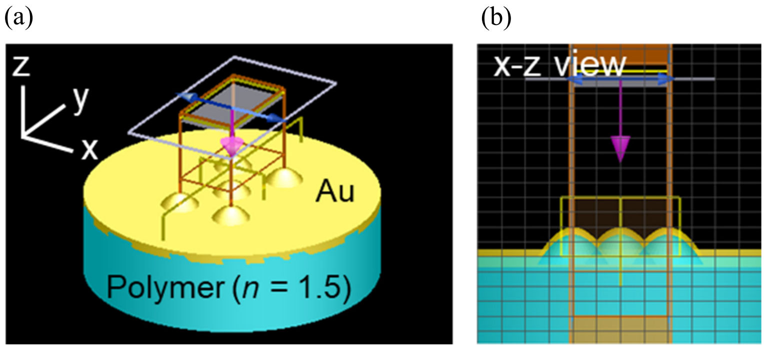

Figure S1. EM simulation using the FDTD method. (a) Perspective view of the simulated model of the Au NCA. The unit cell is located in the x-y plane. (b) x-z view of the simulated model.

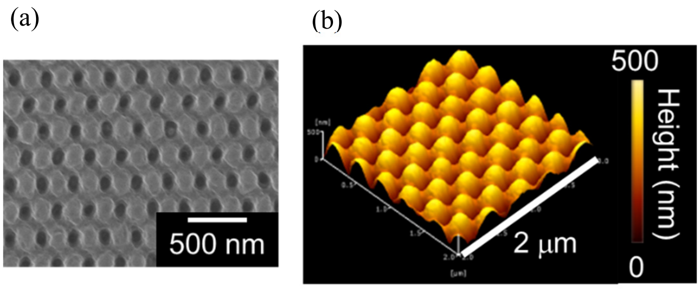

Figure S2. Surface structure of the COP film as observed by (a) FE-SEM and (b) AFM.

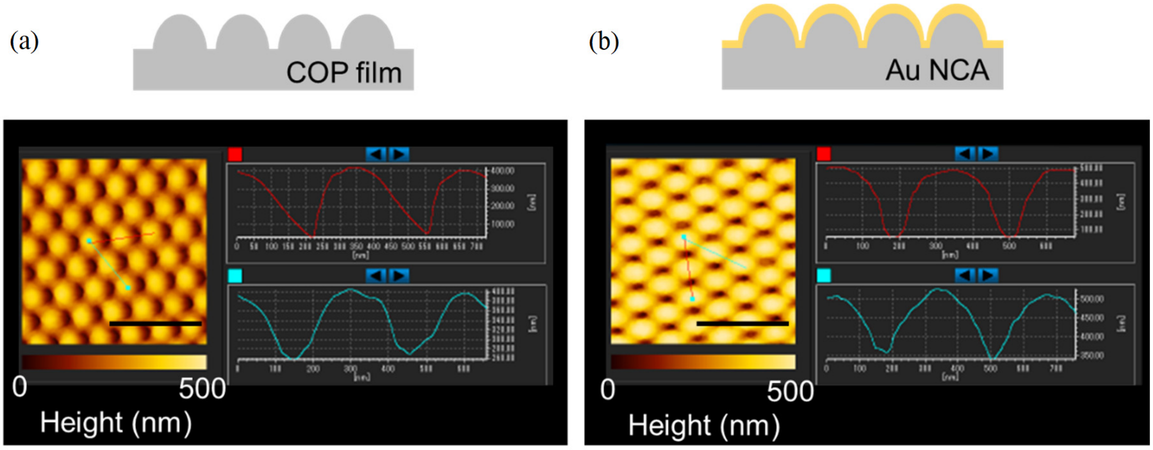

Figure S3. AFM images and cross-section of the surface structure of (a) the COP film and (b) Au NCA. (Black line in AFM images is a 1 μm scale bar).

Evaluation of the individual cone apex radii of the fabricated Au NCA and the COP film

According to Figure S3, we calculated the individual cone apex radii of the fabricated Au NCA and the COP film to confirm the fabrication process reliability.

Then, the individual cone apex radii were calculated by the following steps.

1. The cross-sectional shape numeric data on a blue line (**three cones**) in AFM images (Figure S3a and b) was approximated by a quadratic curve (y = $ax^2 + bx + c$; a, b and c are coefficient).
2. The curvature at the individual cone apex of each cone is approximately determined by $1/2|a|$.

Following the above steps, we calculated the individual cone apex radii. As a result, it was about 74.3 nm (the COP film) and 77.4 nm (the fabricated Au NCA), respectively. The individual cone apex radii of the fabricated Au NCA were larger than the COP film. This is probably caused to use the direct Au deposition on COP film in fabrication process. From this value, we concluded that the fabrication process was mostly trustworthy.

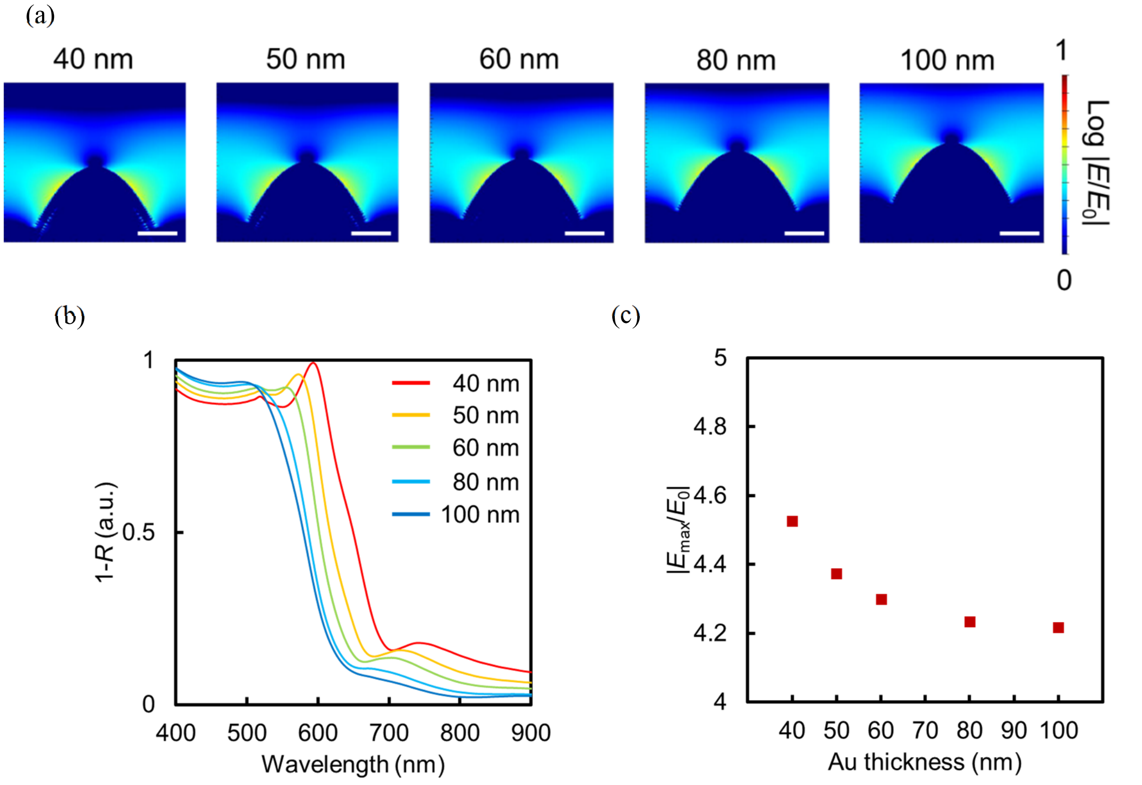

Figure. S4. (a) EM field intensity distribution at 785 nm wavelength (white scale bar is 100 nm), (b) absorption spectra, and (c) maximum value of EM field intensities ($|E_{max}/E_0|$) of Au NCA with various thicknesses of the Au layer.

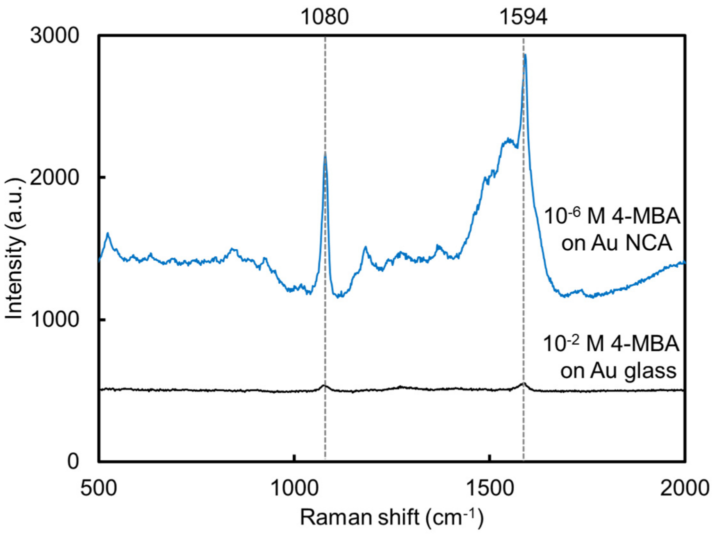

Figure S5. SERS spectra of 10^{-6} M 4-MBA on Au NCA (blue line) and raman spectra 10^{-2} M 4-MBA on Au glass (black line).

Table S1. Band attribution of detected 4-MBA Raman spectra[20, S1–S4]

**Wavenumber (cm^{-1})**	**Band attribution**
722	γ (C-C-C) out of plain vibration
846	δ (COO^-) deformation vibration
1080	ν $(CC)_{ring}$ ring-breathing
1131	δ(CH) deformation vibration
1181	
1589	ν $(CC)_{ring}$ ring-stretching

Calculation of Enhancement Factor (EF)

EF is calculated by the following formula from the literature[46–49];

$$EF = \frac{I_{SERS}}{I_{NR}} \times \frac{N_{NR}}{N_{SERS}} \quad \text{(Equation 1)}$$

where I_{SERS} and I_{NR} correspond to the Raman intensities of 4-MBA on Au NCA and 50 nm Au glass, respectively. N_{SERS} and N_{NR} refer to the number of adsorbed 4-MBA molecules in the laser excitation

area of the Au NCA and 50 nm Au glass, respectively. In addition, N_{SERS} and N_{NR} are expressed by Equations 2 and 3, respectively:

$$N_{SERS} = \frac{\pi\left(\frac{d_{spot}}{2}\right)^2}{S_{Au\ NCA}} \times N_{4\text{-MBA@Au NCA}} \quad \text{(Equation 2)}$$

$$N_{NR} = \frac{\pi\left(\frac{d_{spot}}{2}\right)^2}{S_{Au\ glass}} \times N_{4\text{-MBA@Au glass}} \quad \text{(Equation 3)}$$

where d_{spot}, $S_{Au\ NCA}$, $S_{Au\ glass}$, $N_{4\text{-MBA@Au NCA}}$, and $N_{4\text{-MBA@Au glass}}$ are the diameter of the laser spot, surface area of the fabricated Au NCA, surface area of the Au glass, number of 4-MBA molecules on the Au NCA, and number of 4-MBA molecules on the Au glass, respectively. d_{spot} is expressed as Equation 4.

$$d_{spot} = \frac{1.22 \times \lambda_{ex}}{\text{N.A.}} \quad \text{(Equation 4)}$$

where λ_{ex} and N.A. correspond to the laser wavelength and numerical aperture of the microscope, respectively. d_{spot} was estimated to be 1.20 μm.

For SERS measurements on the Au NCA, a piece of 25 mm^2 Au NCA substrate was used. If all 4-MBA molecules are adsorbed on the Au NCA, N_{SERS} can be calculated as

$$N_{SERS} = \frac{\pi\left(\frac{1.20\ \mu m}{2}\right)^2}{25\ mm^2} \times \left(10^{-6}\ M\right) \times \left(2 \times 10^{-3}\ mL\right) \times N_A = 5.42\times10^7$$

where N_A is the Avogadro constant.

Similarly, in the case of Raman measurement on Au glass, a piece of 64 mm^2 Au glass substrate was used. If all 4-MBA molecules are adsorbed on the Au glass, N_{SERS} can be calculated as

$$N_{NR} = \frac{\pi\left(\frac{1.20\ \mu m}{2}\right)^2}{64\ mm^2} \times \left(10^{-2}\ M\right) \times \left(2 \times 10^{-3}\ mL\right) \times N_A = 1.20\times10^9$$

Consequently, EF is estimated using the two strong peaks at 1080 and 1594 cm^{-1};

$$EF_{1080} = \frac{979.8}{40.35} \times \frac{1.20 \times 10^9}{5.42 \times 10^7} = 9.49 \times 10^4$$

$$EF_{1594} = \frac{1482.3}{50.36} \times \frac{1.20 \times 10^9}{5.42 \times 10^7} = 1.15 \times 10^5$$

References

[S1] Michota, A.; Bukowska, J. Surface-enhanced Raman scattering (SERS) of 4-mercaptobenzoic acid on silver and gold substrates. *J. Raman Spectrosc.* **2003**, 34, 21–25. https://doi.org/10.1002/jrs.928

[S2] Simona, E. H.; Catherine, J. M. Bimetallic silver–gold nanowires: fabrication and use in surface-enhanced Raman scattering. *J. Mater. Chem.* **2006**, 16, 3929–3935. https://doi.org/10.1039/B607116C

[S3] Jun, Y.; Xijiang, H.; Jiaojiao, H.; Leilei, K.; Bin, Z.; Yunchen, D.; Hongtao, Z.; Cunku, D.; Hsing-Lin, W.; Ping, X. Highly Sensitive Surface-Enhanced Raman Spectroscopy (SERS) Platforms Based on Silver Nanostructures Fabricated on Polyaniline Membrane Surfaces. *ACS Appl. Mater. Interfaces* **2012**, 4, 2752−2756. https://doi.org/10.1021/am300381v

[S4] Fortuni, B.; Inose, T.; Uezono, S.; Toyouchi, S.; Umemoto, K.; Sekine, S.; Fujita, Y.; Ricci, M.; Lu, G.; Masuhara, A.; Hutchison, J. A.; Latterini, L.; Uji-I, H. In situ synthesis of Au-shelled Ag nanoparticles on PDMS for flexible, long-life, and broad spectrum sensitive SERS substrates. *Chem. Commun.* **2017**, 53, 11298−11301. https://doi.org/10.1039/C7CC05420C
